# Polymyxin B Resistance in Carbapenem-Resistant *Klebsiella pneumoniae*, São Paulo, Brazil

**DOI:** 10.3201/eid2210.160695

**Published:** 2016-10

**Authors:** Flávia Bartolleti, Bruna Mara Silva Seco, Carla Capuzzo dos Santos, Carolina Bragança Felipe, Mara Elisa Borsato Lemo, Tatiane da Silva Alves, Lilian F. Passadore, Marcelo J. Mimica, Suely Carlos Ferreira Sampaio, Alexandre Prehn Zavascki, Jorge Luiz Mello Sampaio

**Affiliations:** Author affiliations: University of São Paulo School of Pharmaceutical Sciences, São Paulo, Brazil (F. Bartolleti, B.M.S. Seco, J.L. Mello Sampaio);; Santa Casa de São Paulo School of Medical Sciences, São Paulo (F. Bartolleti, M.J. Mimica, S.C.F. Sampaio);; Fleury Medicine and Health, São Paulo (C.C. dos Santos, C.B. Felipe, M.E.B. Lemo, T.S. Alves, J.L.M. Sampaio);; University of São Paulo, São Paulo (L.F. Passadore);; Hospital de Clínicas de Porto Alegre, Porto Alegre, Brazil (A.P. Zavascki)

**Keywords:** carbapenem-resistant Klebsiella pneumoniae, polymyxin B–resistant Klebsiella pneumoniae, polymyxin B resistance, Klebsiella pneumoniae, clonal complex 258, São Paulo, Brazil, bacteria, antimicrobial resistance

**To the Editor:** Infections caused by carbapenem-resistant *Enterobacteriaceae* have been associated with higher death rates than infections caused by carbapenem-susceptible strains, and resistant infections are mostly treated with polymyxins (*1*). Several outbreaks caused by carbapenem- and polymyxin-resistant *Klebsiella pneumoniae* (CPRKp) have been reported, mainly from Europe, and represent an emerging threat.

Carbapenem-resistant *K. pneumoniae* (CRKp) are endemic to Brazil, where polymyxin B (PMB) has been largely used against infections caused by these microorganisms. We evaluated PMB resistance rates and clonal diversity among CRKp isolates from patients in São Paulo, Brazil. The study was approved by the Research Review Board of Fleury Institute in São Paulo.

All *K. pneumoniae* isolates, except those from urine and active surveillance samples, recovered from inpatients during January 1, 2011–December 31, 2015, at 10 private tertiary-care hospitals in São Paulo were included in the study. *K. pneumoniae* isolates were identified by matrix-assisted laser desorption/ionization time-of-flight mass spectrometry; we analyzed only the first isolate from each patient, unless other isolates were recovered after a 90-day interval. To determine antimicrobial drug MICs, we used nonautomated broth microdilution (*2*) polystyrene plates with cation-adjusted Mueller–Hinton broth (Becton Dickinson, Franklin Lakes, NJ, USA) for PMB and tigecycline; the Etest (bioMérieux, Marcy l’Etoile, France) for fosfomycin; and the disk-diffusion method (*3*) for all other antimicrobial drugs. Isolates with a MIC of <2 mg/L for PMB were considered susceptible; this value is the EUCAST (European Committee on Antimicrobial Susceptibility Testing) breakpoint for colistin in *Enterobacteriaceae* (*2*). Tigecycline and fosfomycin MICs were interpreted according to EUCAST guidelines (*2*). Imipenem and meropenem MICs were determined using the Etest for all isolates that were nonsusceptible to at least 1 carbapenem (ertapenem, meropenem, or imipenem) by disk-diffusion (*3*). We phenotypically detected class A carbapenemases as previously described (*4*).

We used convenience sampling to select 62 CPRKp isolates that were detected during 2014–2015 and used pulsed-field gel electrophoresis (PFGE) to evaluate their genomic DNA macrorestriction profiles after *Xba*I digestion. Dice similarity indexes were calculated using the UPGMA method with 1.25% tolerance and optimization (*5*). The minimal Dice index for a clonal group was defined as 80%.

We performed multilocus sequence typing as described (http://bigsdb.web.pasteur.fr/klebsiella/klebsiella.html) for 11 isolates that represented the 2 major PFGE clonal groups, CPRKp1 and CPRKp2. The full *bla*_KPC_ nucleotide sequence was determined for these isolates (Technical Appendix Table 1).

We included a total of 3,085 *K. pneumoniae* isolates in the analysis (Technical Appendix Table 2). A significant increase in carbapenem resistance (p<0.001) was seen from 2011 (6.8%) to 2015 (35.5%) (Figure, panel A). During the last year of analysis, we detected *K. pneumoniae* carbapenemase (KPC) in 96.2% of CRKp isolates.

**Figure:**
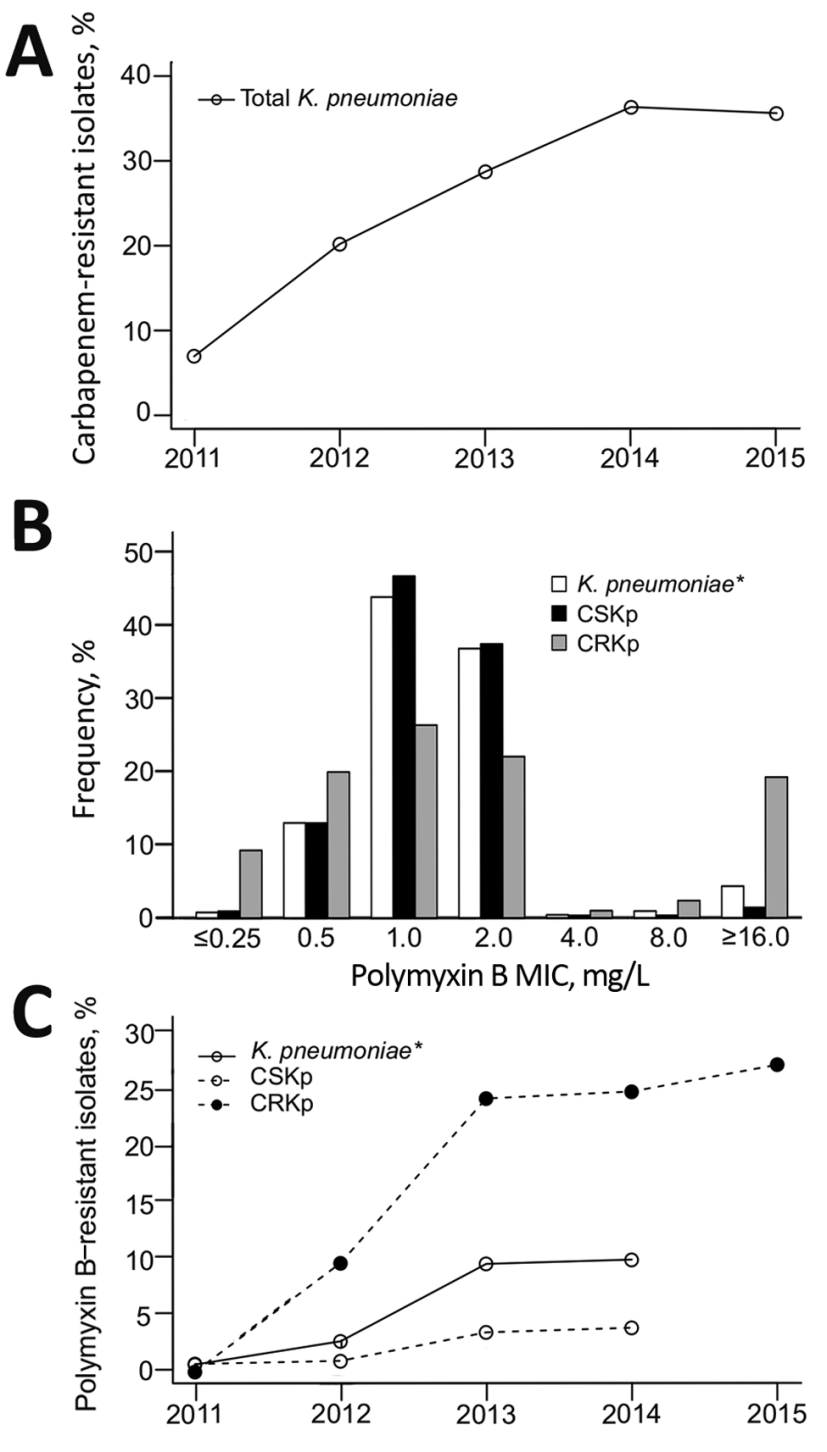
Antimicrobial resistance profile of *Klebsiella pneumoniae* isolated from hospital inpatients in São Paulo, Brazil. A) Carbapenem resistance trend among all *K. pneumoniae* isolates cultured during January 1, 2011–December 31, 2015 (n = 3,085; p<0.001). B) Polymyxin B MIC distribution stratified by carbapenem susceptibility. C) Polymyxin B resistance trend stratified by carbapenem susceptibility, 2011–2015. B, C) Carbapenem-susceptible *K. pneumoniae* (CSKp) isolated during January 1, 2011–June 30, 2014 (n = 1,511) and carbapenem-resistant *K. pneumoniae* (CRKp) isolated during January 1, 2011–December 31, 2014 (n = 436); *during July 1, 2015–December 31, 2015, only CRKp were tested for polymyxin B susceptibility (n = 377). All statistical analyses were conducted using SAS Studio 3.4 (SAS Institute, Inc., Cary, NC, USA). The statistical significance of a trend in resistance rates was evaluated using the χ^2^ test, in which p values <0.05 were considered significant: *K. pneumoniae*, p<0.001; CSKp, p = 0.004; CRKp, p = 0.003.

PMB MICs showed a bimodal distribution that was clearly differentiated by a 2 mg/L MIC (Figure, panel B). When we stratified MICs by year and carbapenem resistance, a significantly increasing trend of resistance was seen among CRKp isolates from 2011 (0%) to 2014 (24.8%) to 2015 (27.1%) (p<0.001) (Figure, panel C). Resistance among carbapenem-susceptible *K. pneumoniae* varied from 0.7% in 2011 to 3.9% in 2014 (p = 0.002).

We did not evaluate the mechanism of PMB resistance. However, this resistance in KPC-producing *K. pneumoniae* is probably caused by the loss of *mgrB* function or the presence of nonsynonymous substitutions in *pmrB* that upregulate the *pmrCAB* and *arnBCADTEF*-*pmrE* operons, resulting in modification of lipid A. All these genes are located on the bacterial chromosome. Susceptibility testing showed that amikacin and tigecycline were the most active non–β-lactam antimicrobial agents against CPRKp isolates (amikacin 73.8%, tigecycline 69.4%) and CRKp isolates (amikacin 79.9%, tigecycline 72.2%) (Technical Appendix Table 3).

PFGE identified 2 major clonal groups. The largest group, CPRKp1 (n = 30), belonged to sequence type (ST) 11, and the other group, CPRKp2 (n = 17), belonged to ST437. Both STs belonged to clonal complex (CC) 258. Interhospital and intrahospital dissemination among private and public hospitals was observed. All isolates tested had the *bla*_KPC-2_ gene (Technical Appendix Figure).

In a previous study, the PMB resistance rate was 27% among 22 CRKp isolates from patients at a tertiary hospital in São Paulo during 2008–2010 (*6*). This rate is much higher than the rate we obtained for 2010, possibly because the previous study had a small number of isolates. CPRKp has been reported in various European countries at rates similar to those we report (*7*).

The predominance of CC258 among KPC-2-producing *K. pneumoniae*, but not among CPRKp, was reported in Brazil (*8*), and ST11, a variant of ST258, has occasionally been detected in colistin-resistant KPC-producing isolates in Spain (*9*). The ST437 clone has been reported in KPC-2 producers in China (*10*), but we found no reports of CPRKp among this clonal group. Our findings show an alarming yearly increase in the rate of PMB resistance among CRKp isolates, mostly KPC-2 producing, and the occurrence of interhospital and intrahospital dissemination of CPRKp from CC258 in São Paulo.

Technical Appendix Supplemental tables and figure for a study of polymyxin B resistance in carbapenem-resistant *Klebsiella pneumoniae* in São Paulo, Brazil.
