# Supplementary material for: Polymyxin B Resistance in Carbapenem-Resistant Klebsiella pneumoniae, São Paulo, Brazil
Source: Emerg Infect Dis. 2016 Oct;22(10):1849–51. doi: 10.3201/eid2210.160695 (PMC5038415; doi:10.3201/eid2210.160695)
Supplement: Technical Appendix — Supplemental tables and figure for a study of polymyxin B resistance in carbapenem-resistant Klebsiella pneumoniae in São Paulo, Brazil. [file 16-0695-Techapp-s1.pdf]

Article DOI: http://dx.doi.org/10.3201/eid2210.160695

Polymyxin B Resistance in Carbapenem-Resistant *Klebsiella pneumoniae*, São Paulo, Brazil

Technical Appendix

Technical Appendix Table 1. Primers used for amplification and sequencing of the *bla*$_{KPC}$ gene

Primer designation	Use	Primer sequence
5-prom-blaKPC-JS	PCR and sequencing	5′-CTAGCTCCACCTTCAAACAAGGA
3-out-blaKPC-JS	PCR and sequencing	5′-TGGGTGGGCCAATAGATGAT
blaKPC-397–416-seqF	Sequencing	5′-GCCGCCAATTTGTTGCTGAA
blaKPC-590–71-seqR	Sequencing	5′-GCAGAGCCCAGTGTCAGTTT

Technical Appendix Table 2. Demographic characteristics of *Klebsiella pneumoniae* isolates included in this study

Characteristic	No. (%) *K. pneumoniae* isolates (n = 3,085)
Year	
2011	468 (15.2)
2012	518 (16.8)
2013	629 (20.4)
2014	662 (21.5)
2015	808 (26.2)
Isolation source	
Blood	1,138 (36.9)
Respiratory tract	892 (28.9)
Other non-urinary non-surveillance	1,055 (34.2)

*During July 1, 2015–December 31, 2015, only carbapenem-resistant *K. pneumoniae* were tested for polymyxin B susceptibility.

Technical Appendix Table 3. Susceptibility of carbapenem-resistant *Klebsiella pneumoniae* and carbapenem and polymyxin B–resistant K. pneumoniae to alternative antimicrobial drugs*

	Susceptibility	
Antimicrobial drug	In CRKp, no. (%)†	In CPRKp, no. (%)†
Amikacin	841 (79.9)	183 (73.8)
Chloramphenicol	530 (23.8)	137 (41.6)
Fosfomycin	258 (27.9)	60 (81.6)
Gentamicin	839 (50.9)	182 (58.2)
Tigecycline	468 (72.2)	111 (69.4)

*CRKp, carbapenem-resistant *K. pneumoniae*. CPRKp, carbapenem- and polymyxin B–resistant *K. pneumoniae*.
†Values indicate susceptibility rate and the number of isolates (n) tested for each antimicrobial drug.

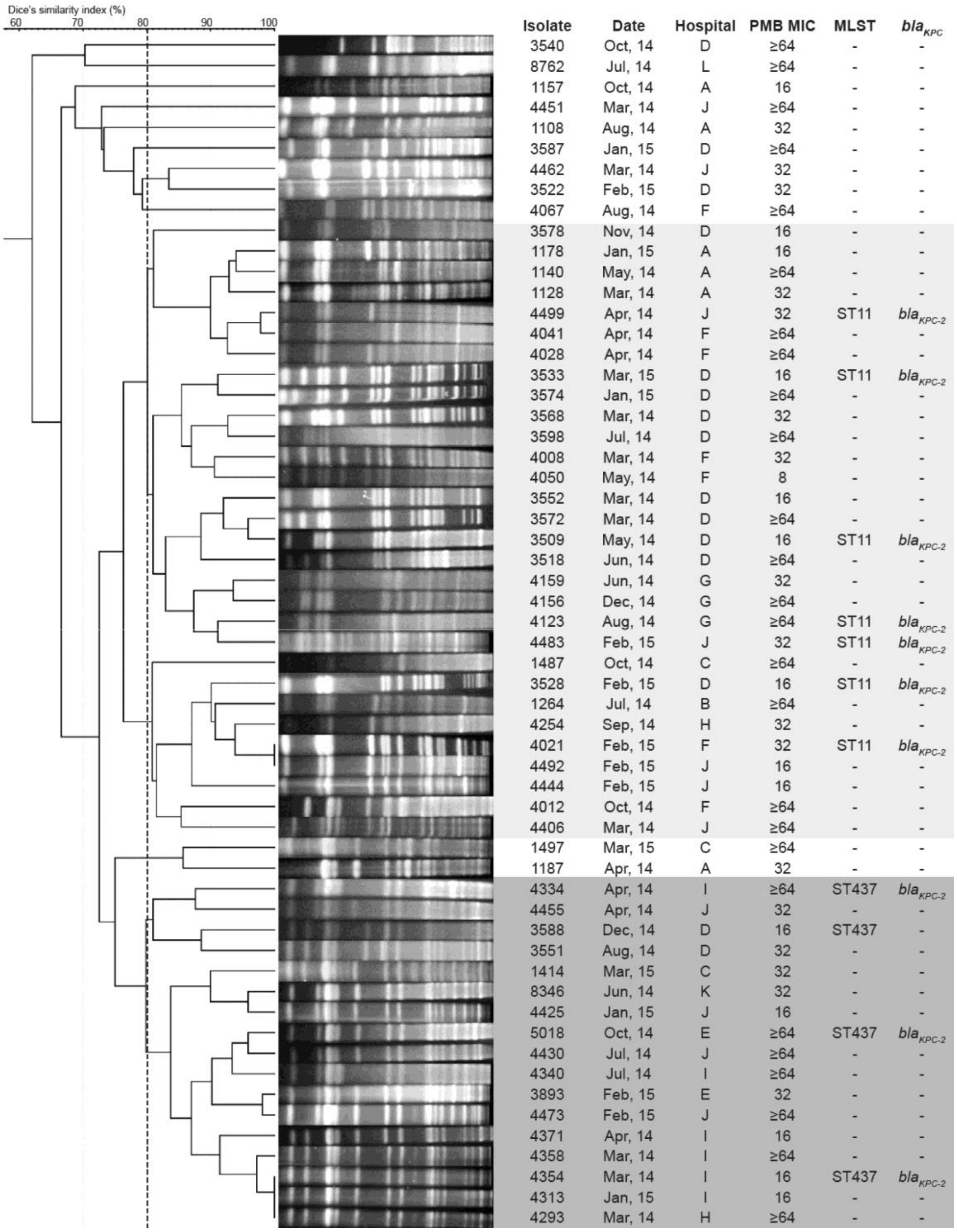

Isolate	Date	Hospital	PMB MIC	MLST	bla_{KPC}
3540	Oct, 14	D	≥64	-	-
8762	Jul, 14	L	≥64	-	-
1157	Oct, 14	A	16	-	-
4451	Mar, 14	J	≥64	-	-
1108	Aug, 14	A	32	-	-
3587	Jan, 15	D	≥64	-	-
4462	Mar, 14	J	32	-	-
3522	Feb, 15	D	32	-	-
4067	Aug, 14	F	≥64	-	-
3578	Nov, 14	D	16	-	-
1178	Jan, 15	A	16	-	-
1140	May, 14	A	≥64	-	-
1128	Mar, 14	A	32	-	-
4499	Apr, 14	J	32	ST11	bla_{KPC-2}
4041	Apr, 14	F	≥64	-	-
4028	Apr, 14	F	≥64	-	-
3533	Mar, 15	D	16	ST11	bla_{KPC-2}
3574	Jan, 15	D	≥64	-	-
3568	Mar, 14	D	32	-	-
3598	Jul, 14	D	≥64	-	-
4008	Mar, 14	F	32	-	-
4050	May, 14	F	8	-	-
3552	Mar, 14	D	16	-	-
3572	Mar, 14	D	≥64	-	-
3509	May, 14	D	16	ST11	bla_{KPC-2}
3518	Jun, 14	D	≥64	-	-
4159	Jun, 14	G	32	-	-
4156	Dec, 14	G	≥64	-	-
4123	Aug, 14	G	≥64	ST11	bla_{KPC-2}
4483	Feb, 15	J	32	ST11	bla_{KPC-2}
1487	Oct, 14	C	≥64	-	-
3528	Feb, 15	D	16	ST11	bla_{KPC-2}
1264	Jul, 14	B	≥64	-	-
4254	Sep, 14	H	32	-	-
4021	Feb, 15	F	32	ST11	bla_{KPC-2}
4492	Feb, 15	J	16	-	-
4444	Feb, 15	J	16	-	-
4012	Oct, 14	F	≥64	-	-
4406	Mar, 14	J	≥64	-	-
1497	Mar, 15	C	≥64	-	-
1187	Apr, 14	A	32	-	-
4334	Apr, 14	I	≥64	ST437	bla_{KPC-2}
4455	Apr, 14	J	32	-	-
3588	Dec, 14	D	16	ST437	-
3551	Aug, 14	D	32	-	-
1414	Mar, 15	C	32	-	-
8346	Jun, 14	K	32	-	-
4425	Jan, 15	J	16	-	-
5018	Oct, 14	E	≥64	ST437	bla_{KPC-2}
4430	Jul, 14	J	≥64	-	-
4340	Jul, 14	I	≥64	-	-
3893	Feb, 15	E	32	-	-
4473	Feb, 15	J	≥64	-	-
4371	Apr, 14	I	16	-	-
4358	Mar, 14	I	≥64	-	-
4354	Mar, 14	I	16	ST437	bla_{KPC-2}
4313	Jan, 15	I	16	-	-
4293	Mar, 14	H	≥64	-	-

Technical Appendix Figure. Clonal diversity among polymyxin B (PMB)– and carbapenem-resistant *Klebsiella pneumoniae* from São Paulo, Brazil. Isolates highlighted in shades of gray represent the 2 largest clonal groups that were detected. Public hospitals are represented by letters A and B. Private hospitals are represented by letters C–L. Date represents the month and year (i.e. 2014, or 2015). MLST, multilocus sequence typing; PMB MIC, PMB MIC in mg/L; ST, sequence type.
